# Structural elucidation of high‐molecular‐weight alkaline degradation products of hexoses

**DOI:** 10.1002/fsn3.1584

**Published:** 2020-05-01

**Authors:** Wen‐Jing Luo, Hai‐Qin Lu, Fu‐Hou Lei, Li‐Yun Cheng, Kai Li, Wen Li

**Affiliations:** ^1^ College of Light Industry and Food Engineering Guangxi University Nanning China; ^2^ Guangxi Key Laboratory of Chemistry and Engineering of Forest Products College of Chemistry and Chemical Engineering Guangxi University for Nationalities Nanning China

**Keywords:** alkaline degradation products of hexoses, high‐molecular‐weight, structural elucidation, sugar industry

## Abstract

High‐molecular‐weight alkaline degradation products of hexoses (HMWHADPs) are colored substances in sugar solutions formed during sugar manufacturing process. These products may be occluded within sugar crystals and impart yellow or brown color to sucrose, thereby negatively affecting the quality of white sugar. Thus, the structural properties of HMWHADPs pose a significant scientific problem in the sugar industry. In the present study, the structural properties of HMWHADPs were investigated using nuclear magnetic resonance, zeta potential analyzer, energy‐dispersive X‐ray spectrometry, ultraviolet–visible spectra, and Fourier transform infrared spectroscopy. Results showed that HMWHADPs mainly contain carboxyl, aldehyde, alcoholic hydroxyl, conjugated double bonds, and saturated alkanes. Possible mechanisms of HMWHADP formation were proposed on the basis of structural property investigation. This study can be used as reference for future research and practice in developing effective methods for the removal of HMWHADPs from sugar solutions and prevention of their further formation in subsequent steps.

## Graphical abstract

The structural properties of high‐molecular‐weight alkaline degradation products of hexoses were investigated using nuclear magnetic resonance, zeta potential analyzer, energy‐dispersive X‐ray spectrometry, and ultraviolet–visible spectra.

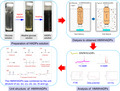

## 1 INTRODUCTION

Alkaline degradation products of hexoses (HADPs) are important colored substances in sugar beet juice, remelt syrup, and sugar cane juice in the sugar industry; they are formed in the sugar manufacturing process as a result of hexose (glucose and fructose) degradation and subsequent polymerization under alkaline and high‐temperature conditions and can be occluded within sugar crystals and impart yellow or brown color to sucrose, thereby negatively affecting the quality of white sugar (Li et al., 2016; Liang, Fan, Zhang, & Song, 2019; Schlumbach, Pautov, & Flöter, 2017; Song, Chai, Zhu, Li, & Liang, 2019). Together with melanoidins, HADPs account for up to 80% of color in sugar beet juices (Coca, García, González, Peña, & García, 2004; Coca, García, Mato, Cartón, & González, 2008). Therefore, the formation of HADPs is a significant problem in the sugar industry. However, the formation process and mechanisms of HADPs are extremely complicated. HADPs are generally divided into two groups, namely low‐ (≤C_6_, LMWHADPs) and high‐molecular‐weight HADPs (>C_6_, HMWHADPs) based on the number of carbon atoms (Coca et al., 2004). The LMWHADPs with six or less carbon are directly degraded from hexoses. LMWHADPs can polymerize to form HMWHADPs which are related to color formation during alkaline degradation reactions. The amounts of HMWHADPs containing more than six carbon atoms may be substantial depending on the conditions of the degradation reactions.

Several studies have been conducted on the alkaline degradation process and products of hexoses (Bruijn, Kieboom, & Bekkum, 1987; Davídek, Robert, Devaud, Vera, & Blank, 2006; Yang & Montgomery, 1996, 2007). The results showed that significantly low‐molecular‐weight organic acids (e.g., formic acid, acetic acid, oxalic acid, lactic acid, and tartaric acid), aldehydes (e.g., formaldehyde, glyceraldehyde, hydroxybutyraldehyde, and glycolaldehyde), and ketones (e.g., methylglyoxal, 1,3‐dihydroxyacetone, and diacetyl) were generated during hexose alkaline degradation reactions. In another study, the molecular weight distribution of HMWHADPs was characterized by gel permeation chromatography (Coca et al., 2004). However, the nature and structure of HMWHADPs have not been elucidated. Coca et al. (2004) considered that HMWHADPs are most likely carboxylic acid products containing more than six carbon atoms. Nevertheless, the empirical evidence available confirming this outcome and elucidating the formation mechanisms of HMWHADPs are limited. To the best of our knowledge, no systematical study reported the nature and structure of HMWHADPs in the field of sugar engineering possibly due to the extremely complex formation process of HMWHADPs in sugar juices and their exceedingly complicated chemical structure. However, the nature and structure of HMWHADPs should be determined to develop suitable technologies that allow their removal from sugar solutions, thereby preventing their further formation in subsequent steps.

In this study, the structural properties of HMWHADPs were investigated using nuclear magnetic resonance (NMR), zeta potential analysis, ultraviolet–visible (UV–vis) spectra, energy‐dispersive X‐ray (EDX) spectrometry, and Fourier transform infrared (FTIR) spectroscopy. This work aimed to contribute fundamentally to the development of suitable technologies for the removal of HMWHADPs from sugar solutions and prevention of their further formation in subsequent steps.

## 2 MATERIALS AND METHODS

### 2.1 HMWHADP preparation

The hexoses mainly include glucose, fructose, mannose, and galactose. Glucose and fructose account for the highest content of hexoses in sugar juices, whereas the contents of other kinds of hexoses are negligible. Coca et al. (2004) summarized and observed that the LMWHADPs degraded from the glucose and fructose are the same. Thus, either glucose or fructose is generally employed as substrate to investigate HADPs in the sugar industry (Coca et al., 2004, 2008; Davídek et al., 2006; Mersad, Lewandowski, Heyd, & Decloux, 2003; Sharma & Johary, 1987; Yang & Montgomery, 2007). In this study, glucose was used to prepare HMWHADPs. Equal volumes of glucose (w/w, 20%) and NaOH solutions (w/w, 5%) were mixed in a conical flask and incubated at 60°C for 1 hr (Zhang, Lin, Cai, & Guo, 2001). Then, the pH of the reaction solution was adjusted to 7.0 ± 0.2 by adding HCl solution. Afterward, the reaction solution was dialyzed by a 20 kDa dialysis bag (SP131348, Shanghai Yuanye Bio‐Technology) to remove the unreacted glucose, LMWHADPs, and other micromolecular intermediate products (Mohsin et al., 2018). In this operation, ultrapure water was used for dialysis; the water was discarded afterward. This step was repeated until the conductivity of the discarded dialysis water was approximately similar to that of ultrapure water, and the unreacted glucose was completely removed from the reaction mixture as determined by high‐performance liquid chromatography. Finally, pure HMWHADPs were obtained after drying by a freeze dryer (Xianou‐12N).

### 2.2 Analysis

The proton NMR (^1^H‐NMR) and carbon‐13 NMR (^13^C‐NMR) spectra of HMWHADPs were obtained with a NMR spectrometer (AVANCE III HD 500, Bruker) by using D_2_O as the solvent and tetramethylsilane as the internal standard. The zeta potentials of the HMWHADP solution under different pH levels were measured by a Zetasizer Nano ZS. The sample pH was adjusted by 0.1 N HCl and 0.1 N NaOH solutions. FTIR spectroscopy (Nicolet iS50, Thermo Fisher Scientific) was used to analyze the functional groups in HMWHADPs. The UV–vis spectra of HMWHADPs were obtained with a spectrophotometer (SPECORD Plus 50). A scanning electron microscope (SUPPRA 55 Sapphire, Carl Zeiss) equipped with an energy‐dispersive X‐ray spectrometer (OXFORD X‐Max 50) was used to analyze the compositions of HMWHADPs.

## 3 RESULTS AND DISCUSSION

### 3.1 Macrocosmic appearance of HADP solution

**FIGURE 1:**
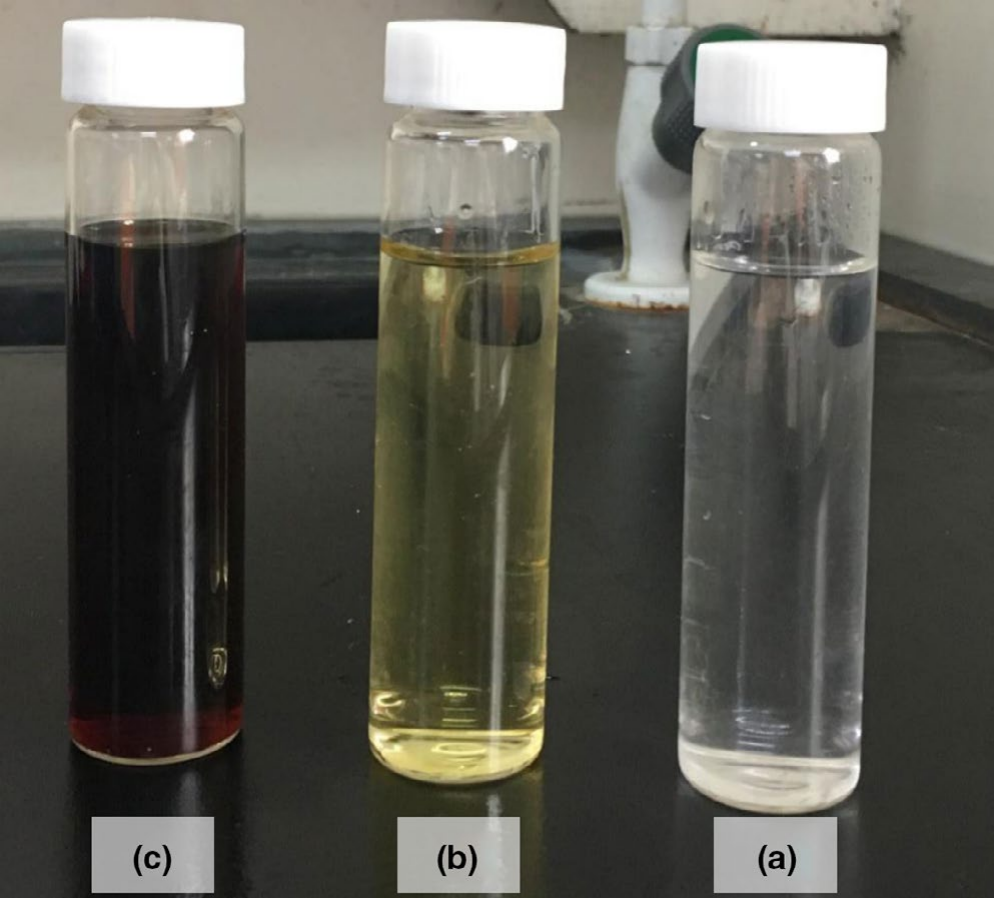
Photograph of glucose (a) and alkaline glucose solutions before (b) and after (c) reaction

Figure 1 shows the photograph of the glucose and alkaline glucose solutions before and after reactions. As shown in Figure 1a, the pure glucose solution was clear and colorless. The alkaline glucose solution showed a pale yellow color before the increase in reaction temperature compared with the pure solution, thereby indicating that hexose degradation easily occurred under alkaline condition. However, the color intensity shown in Figure 1b was considerably lower than that in Figure 1c (HADP solution). Therefore, LMWHADPs can be easily polymerized to form HMWHADPs under alkaline condition.

### 3.2 ^13^C‐NMR analysis

**FIGURE 2:**
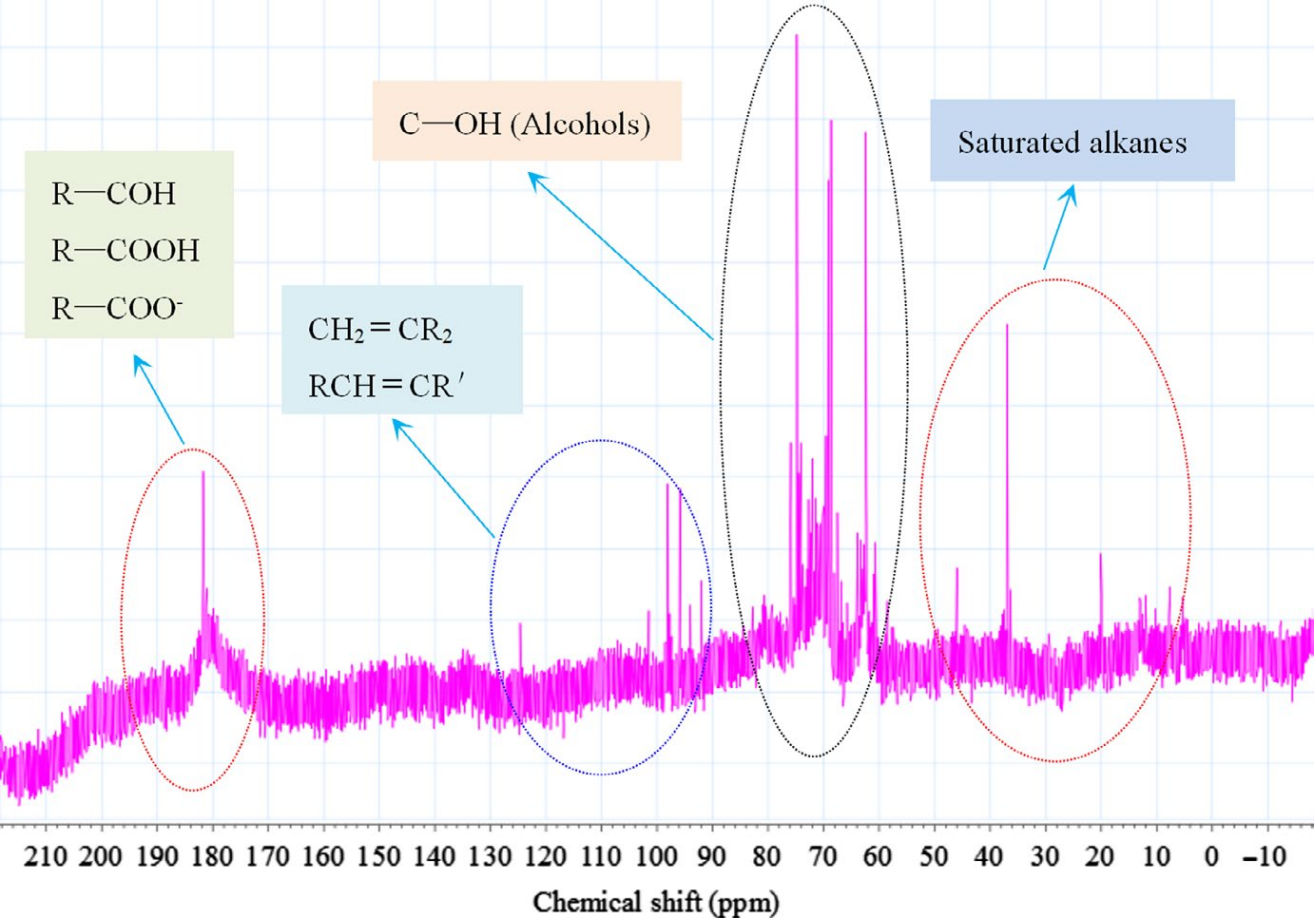
^13^C‐NMR spectra of HMWHADPs

The structural properties of HMWHADPs should be elucidated to provide fundamental information for the development of effective methods for their removal from sugar solutions. Thus, the chemical structure of HMWHADPs was preliminarily characterized by ^13^C‐NMR (D_2_O), and the results are presented in Figure 2. The wide peak regions from 170 to 200 ppm were mainly caused by R–COOH and/or R–COO^−^ (Akhmetov, Gumarov, Petukhov, & Volkov, 2019; Mohsin et al., 2018). A small amount of aldehydes may be included in HMWHADPs. The peaks from 90 to 130 ppm were mainly attributed to unsaturated C=C bonds. The chemical shifts from 60 to 80 ppm were the characteristic peaks of alcohol hydroxyl. The spectra showed peaks from 0 to 50 ppm, which indicated the presence of saturated alkanes in HMWHADPs.

### 3.3 UV–vis analysis

**FIGURE 3:**
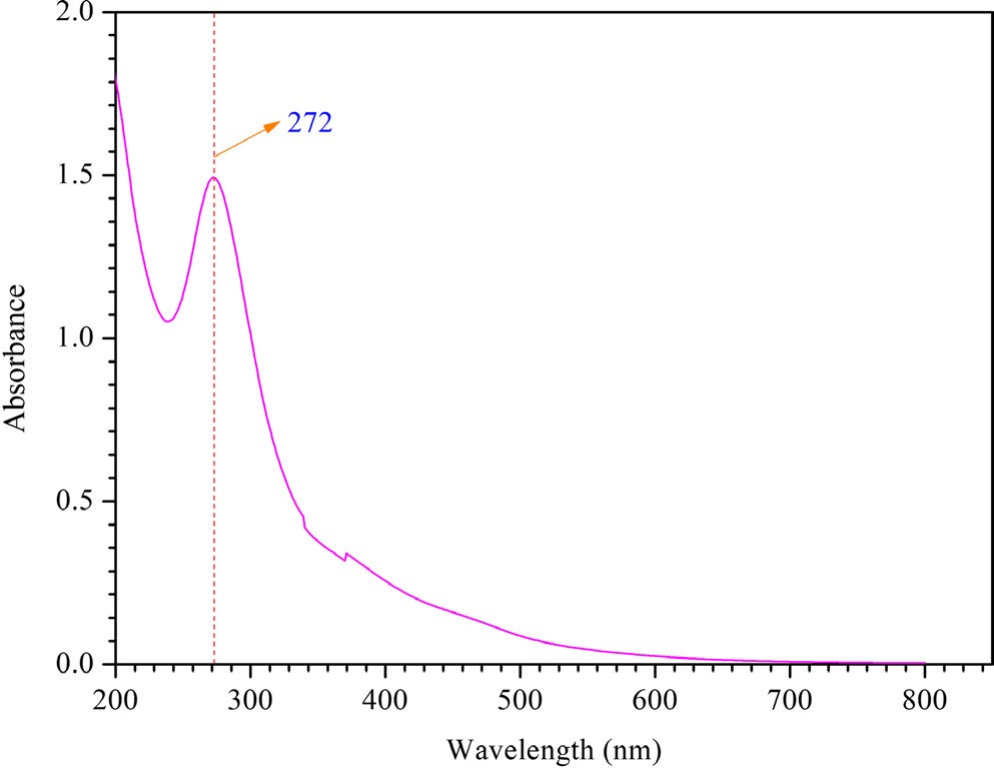
UV–vis spectra of HMWHADPs

**FIGURE 4:**
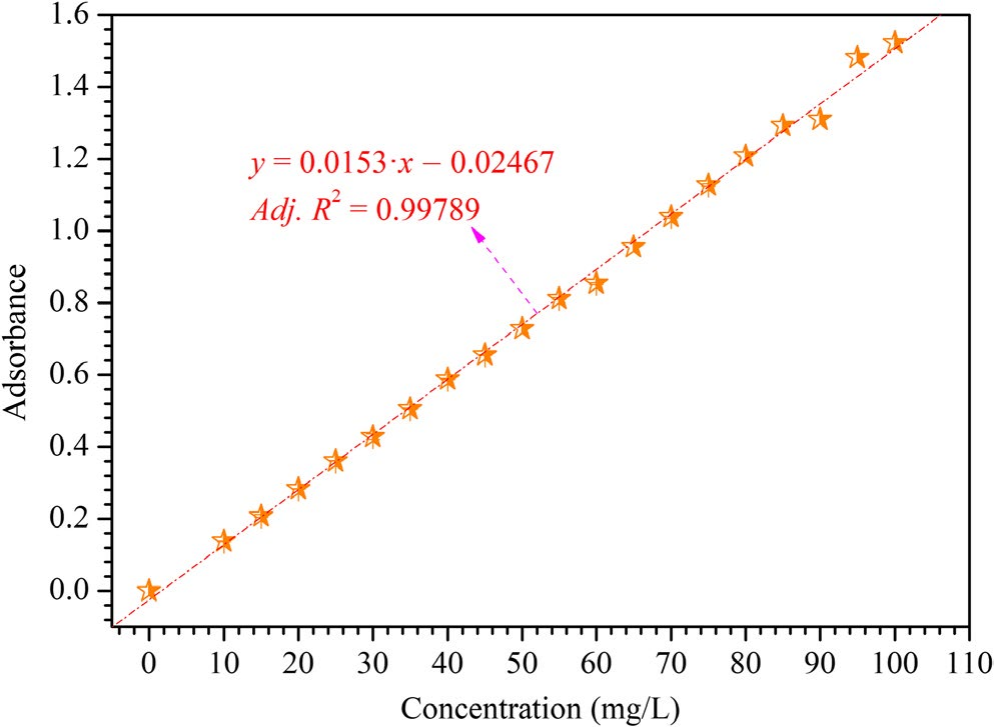
Relationship between HMWHADP concentration and absorbance

To further clarify the structure of unsaturated C=C bonds in HMWHADPs, we characterized HMWHADPs by UV–vis spectra, and the results are shown in Figure 3. The spectra presented a strong absorption peak at 272 nm, which was attributed to the substantial conjugated double bonds in HMWHADPs. The absorbances at 272 nm for the different concentrations of HMWHADP solution were determined, and the results are shown in Figure 4. As shown in Figure 4, a linear relationship existed between the HMWHADP concentration and absorbance (272 nm), and a high adjusted correlation coefficient (Adj.*R* ^2^) exceeding .995 was obtained. These results indicate that the HMWHADP concentration could be determined by UV–vis under its maximum absorption peak (272 nm) in future research and practice.

### 3.4 Zeta potential and ^1^H‐NMR analysis

**FIGURE 5:**
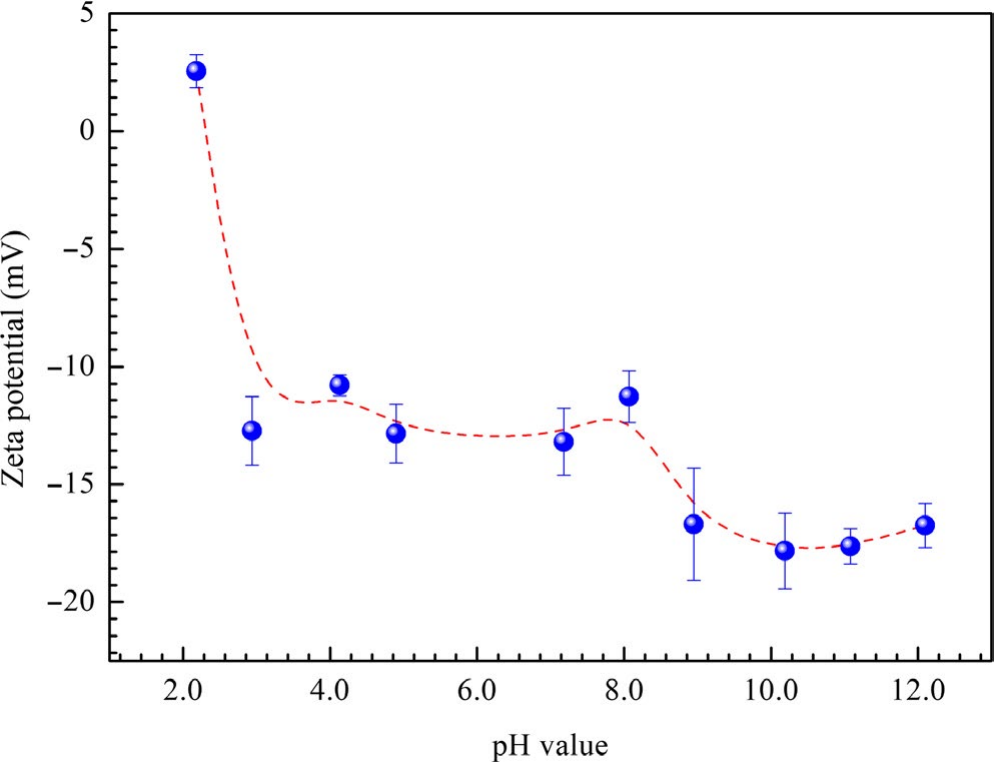
Effects of pH on HMWHADP zeta potential

**FIGURE 6:**
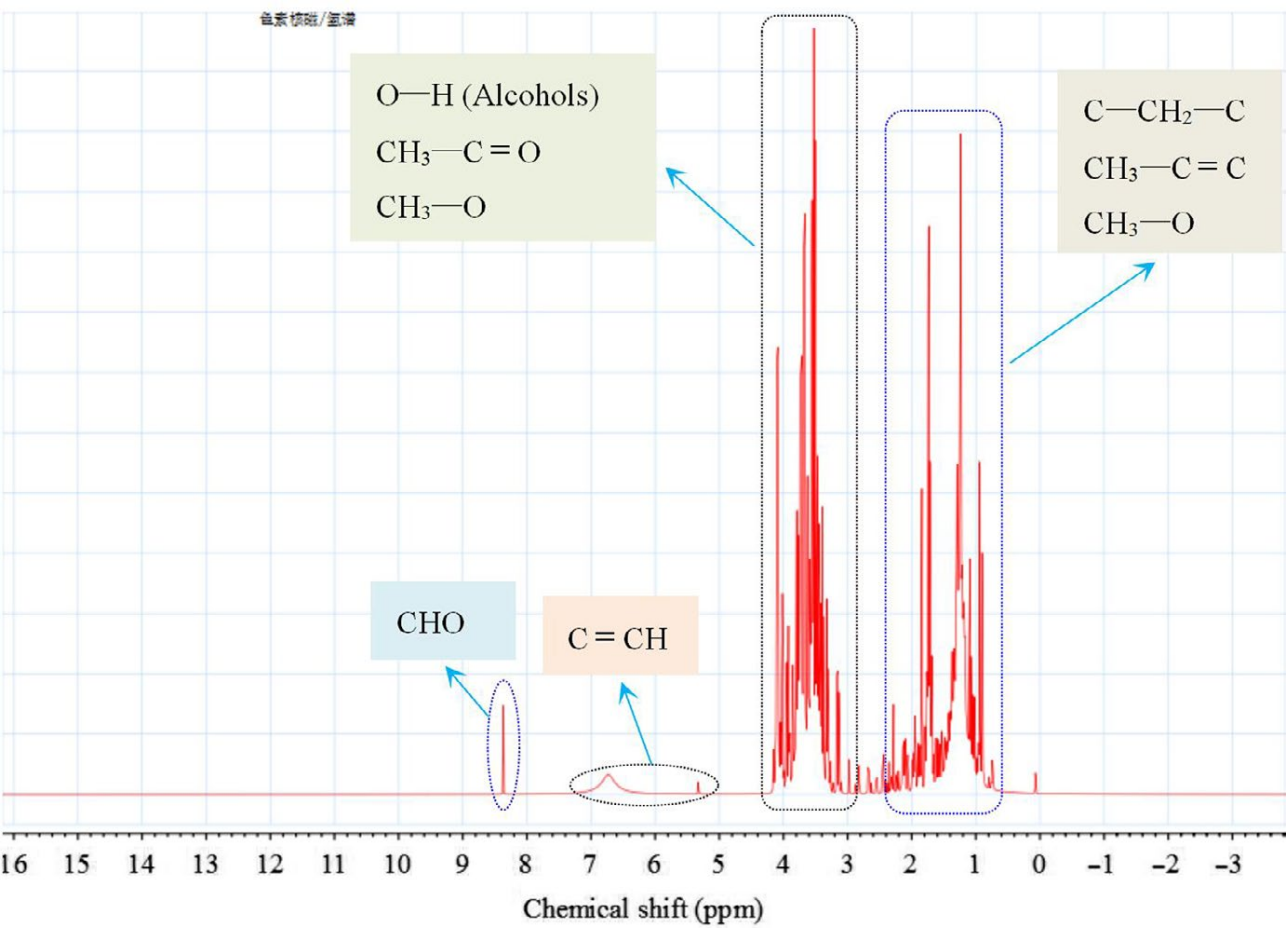
^1^H‐NMR spectra of HMWHADPs

We used a zeta potential analyzer and ^1^H‐NMR (the solvent peaks have been removed automatically by the system) to analyze the HMWHADPs, and the results are presented in Figures 5and6, respectively. As shown in Figure 5, the zeta potential of HMWHADPs decreased with the increase in pH value as a whole. The negative charge of HMWHADPs increased evidently in the pH range of 2–3, indicating that R–COOH and/or R–COO^−^ may be included in HMWHADPs (Huo, 2008). However, no characteristic peaks of R–COOH appeared in the ^1^H‐NMR spectra (Figure 5). This phenomenon can be explained as follows: the dissociation constant for most organic compounds containing a carboxyl group ranges from approximately 10^−2^ to 10^−6^ (pH = 4 ± 2) (Song et al., 2019; Xing, Hu, Chen, & Bai, 2014). During HMWHADP preparation, NaOH was added to the glucose solution to form an alkaline environment. After the reaction, HCl was added to the solution to adjust the pH to approximately 7.0. Finally, HMWHADPs were obtained by dialysis to remove non‐HMWHADP substances. Thus, the carboxyl group in HMWHADPs formed mainly in the form of carboxylate. To further verify this outcome, we used EDX to analyze the compositions of the HMWHADPs, and the results are presented in Table 1. C and O were the major elements of HMWHADPs, whereas low quantities of Na were observed, which verified that carboxyl group in HMWHADPs existed mainly in the form of sodium carboxylate. Several aldehyde groups were also detected in HMWHADPs (Figure 6). The aldehyde content of HMWHADPs was elucidated in subsequent analysis.

**TABLE 1:** EDX data of HMWHADPs

Element	C	O	Na
At (%)	65.64 ± 0.63	30.92 ± 0.93	3.44 ± 0.74

### 3.5 FTIR analysis

**FIGURE 7:**
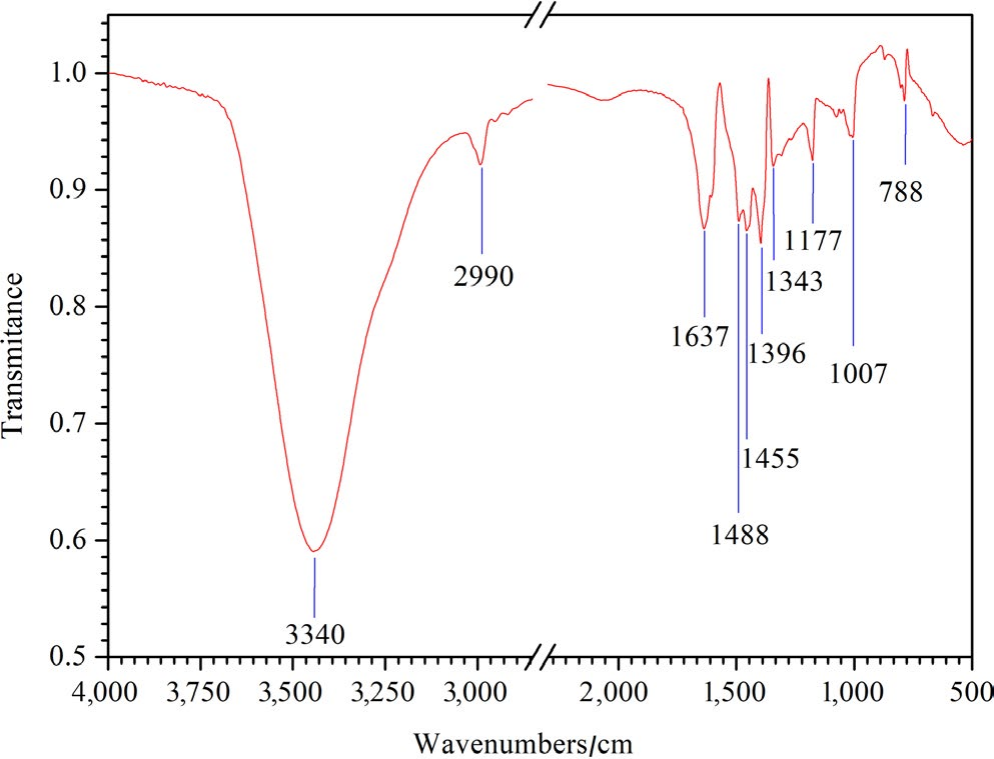
FTIR spectra of HMWHADPs

The HMWHADPs were further investigated by FTIR (Figure 7), and functional groups were conducted to unravel the polymer structure. The typical peak at 3,340 cm^‐1^ was caused by the stretching vibration from hydroxyl (Song et al., 2019). The peak at 2,990 cm^‐1^ was assigned to the C–H group from methylene or methyl (Qu, Li, Hang, Li, & Xie, 2018; Song et al., 2019). The peak at 1,636 cm^‐1^ could be assigned to the C=C and/or C=O bond in HMWHADPs (Dolphen & Thiravetyan, 2011; Mohsin et al., 2018; Simaratanamongkol & Thiravetyan, 2010). The peak at 1,396 cm^‐1^ was attributed to carboxyl and carboxylate (Song et al., 2019). The peak at 1,117 cm^‐1^ was related to the C–O bond in HMWHADPs (Dolphen & Thiravetyan, 2011; Simaratanamongkol & Thiravetyan, 2010). The bands at 788, 1,007, and 1,455 cm^‐1^ were ascribed to C–H and/or –CH_3_ deformation (Dolphen & Thiravetyan, 2011; Mohsin et al., 2018; Simaratanamongkol & Thiravetyan, 2010). The modes at 1,343 and 1,488 cm^‐1^ most probably resulted from O–H in HMWHADPs (Dolphen & Thiravetyan, 2011; Mohsin et al., 2018; Simaratanamongkol & Thiravetyan, 2010). The results obtained from the FTIR spectra corresponded with those of NMR and UV–vis analysis.

## 4 POSSIBLE MECHANISMS FOR HMWHADP FORMATION

Given the investigation and discussion above, the possible formation process of HMWHADPs from hexose (glucose) degradation under alkaline conditions can be described as follows.

Substantial low‐molecular‐weight organic acids, aldehydes, and ketones are generated during hexose alkaline degradation reactions. The aldehydes and a part of ketones readily participate in the aldolization reaction, thereby producing compounds including hydroxyl and aldehyde (Coca et al., 2004). Aldolization products can be converted into olefin aldehyde by dehydration under high temperature. Aldolization is an important organic reaction that leads to the formation of new C–C or C=C bonds and increase in carbon chain. Olefin aldehyde includes two unsaturated double bonds, which feature active chemical properties and can be polymerized to form high‐molecular‐weight compounds. The existence of oxygen in the air in contact with the reaction mixture will accelerate the polymerization reaction and oxidize aldehydes, converting them into carboxyl at the end of the chain in polymerized high‐molecular‐weight compounds (Coca et al., 2004; Huo, 2008; Zhang et al., 2017). Not all aldehydes will be oxidized to form carboxyl. Thus, several aldehydes still exist in HMWHADPs (Figure 6). Nevertheless, further studies are warranted to confirm the possible mechanisms for HMWHADP formation proposed by us.

## 5 CONCLUSIONS

The structural properties of HMWHADPs were investigated using NMR, zeta potential analysis, UV–vis spectra, EDX, and FTIR. Results showed that HMWHADPs mainly contain carboxyl, aldehyde, alcoholic hydroxyl, conjugated double bonds, and saturated alkanes.

## CONFLICT OF INTEREST

The authors declare that they have no conflict of interest.

## ETHICAL APPROVAL

This study did not involve any human or animal testing.
